# Uneven Impact of Maternal Education at Birth on High School Grades of Black and White Students

**DOI:** 10.31586/ojer.2025.1169

**Published:** 2025-02-16

**Authors:** Shervin Assari, Maria Jahromi, Hossein Zare

**Affiliations:** 1 Marginalization-Related Diminished Returns (MDRs) Center, Los Angeles, CA, United States; 2 Department of Family Medicine, Charles R. Drew University of Medicine and Science, Los Angeles, CA, United States; 3 Department of Urban Public Health, Charles R. Drew University of Medicine and Science, Los Angeles, CA, United States; 4 Research School of Economics, Australian National University, Canberra, Australia; 5 School of Economics, University of Sydney, Sydney, Australia; 6 Department of Health Policy and Management, Johns Hopkins Bloomberg School of Public Health, Baltimore, MD, United States; 7 School of Business, University of Maryland Global Campus (UMGC), Adelphi, MD, United States

**Keywords:** Black-White Achievement Gap, Minorities’ Diminished Returns, Maternal Education, High School GPA, Racial Disparities, Structural Racism, Educational Inequality, Fragile Families and Child Wellbeing Study, Social Determinants of Education

## Abstract

**Background:**

The Minorities’ Diminished Returns (MDRs) theory posits that social determinants of health, such as parental education, exert weaker protective effects on health and educational outcomes in racialized and minoritized populations compared to White populations.

**Aim:**

This study examines whether higher maternal education is associated with better high school GPA in Black youth and whether this association aligns with the MDRs framework.

**Methods:**

Data were drawn from the Future of Families and Child Wellbeing Study also known as Fragile Families and Child Wellbeing Study (FFCWS) baseline and 22nd year follow-up (1990–2022). This study included 1873 Black or White participants who were followed from birth to age 22. Linear regression models were used to assess the association between maternal education and high school GPA, adjusting for sociodemographic covariates. Analyses focused on the differential effects of maternal education across racial groups, particularly among Black youth.

**Results:**

While maternal education was positively associated with high school GPA, this effect was weaker for Black students compared to their White counterparts. Specifically, each additional year of maternal education corresponded to a lower GPA increase in Black students, consistent with the MDRs hypothesis.

**Conclusion:**

Findings support the MDRs theory, indicating that maternal education has a reduced protective effect on high school GPA among Black youth. These results underscore the need for policies that address structural factors beyond education to promote equitable academic achievement.

## 1. Introduction

The Black-White achievement gap has persisted as a significant contributor to broader racial disparities in economic and health outcomes [1,2]. Traditionally, this gap has been attributed to factors such as lower socioeconomic status (SES) and under- resourced schools in predominantly Black communities [3,4]. While these inequalities undoubtedly play a role, recent frameworks suggest a more nuanced picture that involves the differential effectiveness of these resources across racial groups, due to structural and historical racism [5].

Educational attainment in childhood and adolescence is a well-documented predictor of future success, influencing not only academic outcomes but also cognitive, behavioral, economic, and health trajectories [6–13]. Academic performance in youth, often reflected in grade point average (GPA), serves as both a marker of high school success and a crucial determinant of future educational and career opportunities. Youth with lower academic achievement frequently face obstacles in gaining college admission, are more likely to enter the labor market with limited qualifications and may be restricted to lower-paying jobs [13]. As a result, the Black-White achievement gap in GPA and overall academic performance has far-reaching implications, contributing to enduring disparities in economic stability and health that persist into adulthood.

One of the most influential factors shaping youth academic outcomes, particularly GPA, is maternal education [14–18]. Children and youth from families with higher maternal education levels generally have access to enhanced academic support and resources, translating into improved GPA and overall school performance [19–22]. In contrast, children with parents who have lower educational attainment are more likely to experience academic difficulties, often resulting in lower GPA [23–25]. Some of the observed Black-White gap in GPA has been attributed to generally lower parental education levels among Black families, reflecting generational effects of educational and economic disadvantages [26,27].

However, the Minorities’ Diminished Returns (MDRs) framework [28,29] provides an alternative perspective by positing that the benefits of maternal education are not equally distributed across racial groups. MDRs suggest that while maternal education can improve academic performance, Black youth receive a comparatively smaller benefit from maternal educational attainment than their White peers [30,31]. This framework implies that even with higher levels of maternal education, Black youth face structural barriers— such as discrimination in schools, fewer resources, and higher levels of neighborhood disadvantage—that can dampen the positive effects of maternal education on academic success [32–35].

The present study leverages data from the Fragile Families and Child Wellbeing Study (FFCWS) [36–38] to examine whether maternal education is positively associated with high school GPA and whether this association is moderated by race. By analyzing the differential impact of maternal education on the GPA for Black and White youth, this research aims to deepen our understanding of the complex role that structural racism plays in shaping academic outcomes for Black students. This study also highlights potential policy interventions that could help reduce the achievement gap by addressing the structural factors that limit the effectiveness of maternal education for Black youth.

We hypothesize that maternal education will be positively associated with high school GPA, but that this association will be weaker for Black youth than for White youth, consistent with the MDRs framework. Findings from this study are expected to align with MDRs, suggesting that addressing broader structural inequities may be necessary to ensure that Black youth fully benefit from parental educational attainment and achieve equitable educational outcomes.

## 2. Methods

### Study Design and Sample:

This study utilizes data from the Fragile Families and Child Wellbeing Study (FFCWS) [36–38], a longitudinal study tracking a cohort of children born in large US cities between 1998 and 2000, primarily to unmarried parents. The FFCWS data includes information on family structure, socioeconomic status, and various child and family outcomes over a 22- year period, providing a rich source for investigating long-term academic outcomes.

The Fragile Families and Child Wellbeing Study (FFCWS) [36–38] offers a unique and valuable resource for examining racial and socioeconomic disparities in child development and outcomes across the life course. Launched in the late 1990s, the FFCWS follows a cohort of nearly 5,000 children born in large U.S. cities, with an intentional oversampling of children born to unmarried parents, providing comprehensive data on families often underrepresented in other studies. The study’s longitudinal design, spanning over two decades, allows researchers to track and analyze long-term outcomes, such as educational attainment, health, and socioeconomic status, from birth into young adulthood. The rich demographic, socioeconomic, and behavioral data collected through extensive surveys and interviews capture both family and community-level factors, enabling a nuanced understanding of how variables like parental education interact with structural factors to impact outcomes across racial groups. Furthermore, the FFCWS provides an opportunity to explore the mechanisms underlying theories like Minorities’ Diminished Returns (MDRs) [39–45], as it offers both a racially diverse sample and repeated measures that allow for examining changes over time. These features make FFCWS uniquely suited for studying disparities and the complex social determinants of health and educational outcomes in marginalized populations.

### Analytical Sample:

This study included 1,873 Black or White participants who were followed from birth to age 22. Participants were those who had data on high school average grades at age 22 (self-report) and identified self as either White or Black at age 22.

### Outcome:

#### High School GPA:

Average high school grades (GPA), the primary outcome, was self-reported by participants during the 22-year follow-up. The question was: “What best represents your grades in high school?” Responses included Mostly A’, About half A’s and B’s, Mostly B’s, About half B’s and half C’s, Mostly C’s, About half D’s and half C’s, Mostly D’s, and Mostly below D’s (1–8). Higher score indicated higher GPA.

### Measures:

#### Race:

Self-identified by the child at age 22, race was a dichotomous variable coded 1 for Black and 0 for White.

#### Poverty Level (Baseline).

Poverty level at baseline is measured using the income-to- needs ratio, based on the federal poverty rate, as reported by the mother at the time of the child’s birth. This measure is derived from household income data collected during the baseline wave and subsequent follow-ups of the Fragile Families and Child Wellbeing Study. Families are classified according to the federal poverty threshold, with the income- to-needs ratio indicating whether the household falls below, at, or above the poverty line. This variable serves as a key indicator of early-life socioeconomic disadvantage, which has been associated with various developmental, health, and educational outcomes throughout childhood and adolescence. The poverty level variable ranges from 0 to 14, with higher values indicating higher socioeconomic status (SES).

#### Born from an Adulthood Pregnancy (Baseline).

This variable indicates whether the child was born to a mother who was at least 18 years old at the time of delivery, distinguishing between adolescent and adult pregnancies. The Fragile Families and Child Wellbeing Study collects maternal age at childbirth, allowing researchers to differentiate between children born to teenage mothers (under 18) and those born to adult mothers (18 and older). This distinction is relevant for understanding potential differences in early childhood environments, parental resources, and long-term developmental outcomes, as children born to younger mothers may face heightened socioeconomic and educational disadvantages.

#### Born Low Birth Weight (Baseline).

Low birth weight (LBW) is defined as a birth weight of less than 2,500 grams (5.5 pounds), a threshold established by the World Health Organization (WHO) and commonly used in public health research. In the FFCWS, birth weight data is collected from medical records or maternal reports at the baseline interview. LBW is a key indicator of early-life health risks and has been associated with adverse developmental, cognitive, and health outcomes in childhood and beyond. It is also a critical factor in studying health disparities, as racial, socioeconomic, and environmental inequalities contribute to variations in birth weight across different populations.

#### Maternal Education:

Assessed at baseline of the FFCWS, maternal education was measured as the highest grade or degree completed by the mother. This variable was an ordinal variable ranging from 1 to 4 (less than high school diploma, high school diploma, some college, and college graduate).

### Covariates:

Key sociodemographic covariates included child age, gender, low birth weight, born to teen pregnancy (at baseline), father incarceration (at baseline), poverty level (at baseline), and family structure (at baseline). These variables were controlled to account for variations in socioeconomic conditions.

### Analytic Approach:

To test the hypothesis that maternal education’s effect on GPA varies by race, we applied a linear regression model (ordinary least squares model) predicting high school GPA as a function of maternal education, race, and the interaction between maternal education and race. This interaction term captures whether the association between maternal education and GPA differs for Black youth relative to other racial groups. Models adjusted for all aforementioned covariates. Analysis was conducted using Stata 18.0, with a significance threshold set at p < 0.05.

## 3. Results

The descriptive statistics for the study sample are presented in Table 1. The total sample size for the study was 1,873. In terms of racial distribution, the sample predominantly consisted of Black participants, who represented 71.81% (n = 1,345) of the cohort, compared to 28.19% (n = 528) who were White. The gender distribution was relatively balanced, with females comprising 52.75% (n = 988) and males 47.25% (n = 885) of the sample. Regarding father incarceration status at baseline, a majority of participants (96.21%) reported no history of paternal incarceration, while 3.79% (n = 70) had a father who was incarcerated. Maternal education levels varied, with 25.98% (n = 486) of mothers having the lowest level of education (less than a high school diploma), 33.24% (n = 622) having a low level (a high school diploma), 25.82% (n = 483) having a medium level (some college), and 14.97% (n = 280) achieving a high level of education (college graduate). Teen pregnancy was notably rare, with 91.03%, only 8.97% (n = 168) were born to teen mothers and 1,704 of participants born to mothers who had a pregnancy during their adulthood. Low birth weight was reported for 10.09% (n = 189) of the sample, while the majority (87.03%, n = 1,630) had a birth weight within the normal range.

Family structure at birth showed that a substantial proportion of children (74.27%, n= 1,391) were born to unmarried parents, whereas 25.73% (n = 482) were born to married parents.

The mean baseline poverty level, measured on a scale from 0 to 14, was 2.55 (standard error = 0.06). The mean school performance score, assessed on a scale from 1 to 8, was 5.86 (standard error = 0.03).

The results from the model without interaction are summarized in Table 2. Race (Black) was not significantly associated with high school performance (coefficient = −0.098, standard error = 0.086, 95% confidence interval [CI]: −0.267 to 0.070, p = 0.253). Gender was significantly associated with high school performance, with male students having lower performance scores than females (coefficient = −0.531, standard error = 0.066, 95% CI: - 0.660 to −0.403, p < 0.001). Low birth weight at baseline did not significantly predict high school performance (coefficient = 0.005, standard error = 0.033, 95% CI: −0.061 to 0.070, p =0.889).

Being born to a married family was significantly associated with higher high school performance (coefficient = 0.207, standard error = 0.096, 95% CI: 0.020 to 0.395, p = 0.030). The mother’s poverty ratio was also a significant positive predictor of high school performance (coefficient = 0.088, standard error = 0.016, 95% CI: 0.056 to 0.121, p < 0.001). Father’s incarceration status at baseline showed no significant association with high school performance (coefficient = 0.002, standard error = 0.172, 95% CI: −0.336 to 0.340, p =0.991).

The maternal age at the time of pregnancy (indicating adulthood pregnancy) was also non-significant (coefficient = −0.033, standard error = 0.120, 95% CI: −0.267 to 0.202, p= 0.784). However, maternal education at baseline was positively associated with high school performance, indicating that higher maternal education predicted higher performance scores (coefficient = 0.186, standard error = 0.042, 95% CI: 0.104 to 0.269, p <0.001).

### Outcome: High School Performance (1–8)

The results from the interaction model in Table 3 indicate that maternal education at baseline was positively associated with high school performance, suggesting that higher maternal education predicted higher performance scores (coefficient = 0.335, standard error = 0.068, 95% CI: 0.201 to 0.468, p < 0.001). However, the interaction term between maternal education and race (Black) was significant and negative, indicating that the positive effect of maternal education on high school performance was weaker for Black youth compared to White youth (coefficient = −0.207, standard error = 0.075, 95% CI: −0.354 to −0.060, p = 0.006). In addition, being born to a married family was marginally associated with higher high school performance, though not statistically significant at the conventional threshold (coefficient = 0.165, standard error = 0.097, 95% CI: −0.024 to 0.354, p = 0.088). The mother’s poverty ratio remained a significant positive predictor of high school performance (coefficient = 0.083, standard error = 0.017, 95% CI: 0.050 to 0.115, p < 0.001). Father’s incarceration status at baseline was not significantly associated with high school performance (coefficient = −0.019, standard error = 0.172, 95% CI: −0.356 to 0.319, p= 0.914). Gender showed a significant effect, with male students having lower performance scores than female students (coefficient = −0.538, standard error = 0.065, 95% CI: −0.666 to −0.409, p < 0.001). Being born with low birth weight did not significantly predict high school performance (coefficient = 0.012, standard error = 0.033, 95% CI: −0.054 to 0.077, p = 0.722). The maternal age at pregnancy (adulthood pregnancy) was also non- significant (coefficient = −0.028, standard error = 0.119, 95% CI: −0.262 to 0.206, p = 0.812).

### Outcome: High School Performance (1–8)

## 4. Discussion

The primary aim of this study was to assess the association between maternal education and high school GPA and to determine if this relationship differed by race, specifically comparing Black and White youth. We hypothesized that higher maternal education would generally correlate with better academic performance, as measured by GPA, but that the strength of this association would be weaker for Black youth than for White youth, consistent with the Minorities’ Diminished Returns (MDRs) framework. Our findings confirmed both the main effect—maternal education positively influencing GPA—and the interaction effect, demonstrating that Black youth experience fewer academic benefits from maternal education than their White counterparts.

The results underscore the MDRs framework by highlighting that the positive association between maternal education and high school GPA is attenuated for Black youth, even when controlling for relevant covariates. While maternal education generally enhances academic outcomes, Black students gain less from their mothers’ educational attainment compared to White students. This discrepancy likely reflects enduring structural barriers such as school segregation, discriminatory educational policies, and fewer academic resources, all of which disproportionately impact Black students regardless of family background. These findings suggest that addressing the academic achievement gap may require more than simply increasing maternal education; it necessitates a deeper engagement with the social and structural factors that differentially constrain Black youth’s educational outcomes.

Our first key finding emphasizes that increasing maternal education can serve as a vital strategy to improve youth academic achievement. Higher maternal education often translates into a more supportive home environment, access to higher-quality schools, greater parental involvement in school-related activities, and better support for homework and learning [18,46–48]. Additionally, children of more educated mothers may develop higher aspirations and a stronger commitment to academic success [49–53]. Importantly, investments in parental education yield multigenerational benefits, as the advantages of higher maternal education extend into the next generation. Thus, policies that facilitate educational access and attainment for parents may serve as a long-term investment in future academic success for their children.

Our second key finding indicates that increasing maternal education alone is unlikely to be sufficient to close the academic achievement gap between Black and White students. This finding is in line with previous findings in other studies [31,34,54–58]. Structural barriers must be addressed to create equitable educational opportunities that allow Black youth to fully benefit from their parents’ educational achievements. Policy efforts are needed to tackle resource disparities in schools serving predominantly Black communities, combat discrimination within educational settings, and address broader economic inequalities that hinder Black families’ overall socioeconomic mobility [59–61]. By acknowledging these structural challenges, policymakers and educators can more effectively promote policies that level the playing field for Black youth and enhance the returns of maternal education across racial groups.

A key strength of this study was the unique dataset utilized. We leveraged data from the Fragile Families and Child Wellbeing Study (FFCWS), which offers a rare opportunity to examine the mechanisms underlying theories such as Minorities’ Diminished Returns (MDRs) [39–45]. The dataset’s racially diverse sample and repeated measures enable the assessment of disparities and changes over time, making it particularly well-suited for studying the social determinants of health and educational outcomes in marginalized populations. Unlike more nationally representative samples, where White children often come from highly privileged backgrounds, FFCWS includes White children from less advantaged families, providing a more appropriate comparison group for understanding how socioeconomic resources translate into outcomes across racial groups. This distinct advantage strengthens the study’s contribution to the literature by demonstrating that MDRs are not only evident in nationally representative datasets but also among low- income, unwed, and fragile families. These findings have important implications for educational policy. Policies should go beyond promoting individual educational attainment and address the broader structural inequities that limit the effectiveness of such educational gains for Black families. Resource allocation should prioritize underfunded schools in predominantly Black communities, with efforts to improve teacher support, learning materials, and infrastructure. Anti-discrimination measures are also essential to create safe and inclusive academic environments where Black students can thrive and capitalize on parental resources. Comprehensive policy efforts that recognize the complexity of educational inequalities and support both individual and structural interventions could play a critical role in reducing the achievement gap.

Despite these significant contributions, this study has some limitations. First, it relies on self-reported GPA, which may be subject to reporting biases. Second, while we controlled for several important covariates, unmeasured factors, such as neighborhood quality, school resources and peer influences, could also affect academic outcomes. In addition, our outcome (GPA) was an ordered categorical variable. Further replications and sensitivity analyses using alternative techniques and approaches are needed to strengthen confidence in our results. For example, in the field of economics, researchers may analyze the same data using an ordered logit or probit model to validate and extend our findings. Finally, as this study focuses on Black and White youth, future research should examine similar patterns among other racial and ethnic groups to understand how MDRs might manifest across different populations.

## 5. Conclusion

In summary, this study contributes to the growing body of evidence supporting the Minorities’ Diminished Returns framework, particularly in the context of educational outcomes. While maternal education is generally associated with higher academic achievement, Black youth derive fewer benefits from maternal educational attainment than White youth, underscoring the role of structural racism in shaping educational inequalities. Policies that aim to close the achievement gap must go beyond promoting parental education alone and address the broader structural conditions that limit Black youth’s academic opportunities. By investing in equitable educational resources, anti- discrimination policies, and supportive school environments, we can work toward a future in which all students have the opportunity to reach their full academic potential.

## Figures and Tables

**Table 1.** Descriptive Data, Overall (N=1,873)

	Mean	Std. err.
Baseline Mother Poverty to Income Ratio (0–14)	2.55	0.06
School Performance (1–8)	5.86	0.03
		
	**n**	**%**
Race		
White	528	28.19
Black	1,345	71.81
Gender		
Female	988	52.75
Male	885	47.25
Father Incarceration		
No	1,776	96.21
Yes	70	3.79
Maternal Education		
Lowest (Below high school diploma)	486	25.98
Low (High school diploma)	622	33.24
Medium (Some college)	483	25.82
High (College graduate)	280	14.97
Born to Adulthood Pregnancy		
No	168	8.97
Yes	1,704	91.03
Low Birth Weight		
No	1,630	87.03
Yes	189	10.09
Family Structure (Birth)		
Unmarried	1,391	74.27
Married	482	25.73

**Table 2.** Model without interaction

					
	Coefficient	Std. Err.	[95% conf.	interval]	P>t
					
Race (Black)	−0.098	0.086	−0.267	0.070	0.253
Gender (Male)	−0.531	0.066	−0.660	−0.403	< 0.001
Born Low Birth Weight (Baseline)	0.005	0.033	−0.061	0.070	0.889
Born to Married Family	0.207	0.096	0.020	0.395	0.030
Baseline Mother Poverty to Income Ratio	0.088	0.016	0.056	0.121	< 0.001
Father Incarceration (Baseline)	0.002	0.172	−0.336	0.340	0.991
Born from an Adulthood Pregnancy (Baseline)	−0.033	0.120	−0.267	0.202	0.784
Maternal Education (Baseline)	0.186	0.042	0.104	0.269	< 0.001
Intercept	5.485	0.151	5.189	5.781	< 0.001

Outcome: High School Performance (1–8)

**Table 3.** Model with interaction

					
	Coefficient	Std. Err.	[95% conf.	interval]	P>t
					
Race (Black)	0.419	0.205	0.016	0.822	0.042
Gender (Male)	−0.538	0.065	−0.666	−0.409	< 0.001
Born Low Birth Weight (Baseline)	0.012	0.033	−0.054	0.077	0.722
Born to Married Family	0.165	0.097	−0.024	0.354	0.088
Baseline Mother Poverty to Income Ratio	0.083	0.017	0.050	0.115	< 0.001
Father Incarceration (Baseline)	−0.019	0.172	−0.356	0.319	0.914
Born from an Adulthood Pregnancy (Baseline)	−0.028	0.119	−0.262	0.206	0.812
Maternal Education (Baseline)	0.335	0.068	0.201	0.468	< 0.001
Maternal Education (Baseline) x Race (Black)	−0.207	0.075	−0.354	−0.060	0.006
Intercept	5.106	0.204	4.706	5.505	< 0.001

Outcome: High School Performance (1–8)
